# Discovery of the Corallivorous Polyclad Flatworm, *Amakusaplana acroporae*, on the Great Barrier Reef, Australia – the First Report from the Wild

**DOI:** 10.1371/journal.pone.0042240

**Published:** 2012-08-01

**Authors:** Kate A. Rawlinson, Jessica S. Stella

**Affiliations:** 1 Biology Department, Dalhousie University, Halifax, Nova Scotia, Canada; 2 Australian Research Council Centre of Excellence for Coral Reef Studies, and School of Marine and Tropical Biology, James Cook University, Townsville, Queensland, Australia; 3 Climate Adaptation Flagship, Commonwealth Scientific and Industrial Research Organisation, Hobart, Tasmania, Australia; Leibniz Center for Tropical Marine Ecology, Germany

## Abstract

The role of corallivory is becoming increasingly recognised as an important factor in coral health at a time when coral reefs around the world face a number of other stressors. The polyclad flatworm, *Amakusaplana acroporae*, is a voracious predator of Indo-Pacific acroporid corals in captivity, and its inadvertent introduction into aquaria has lead to the death of entire coral colonies. While this flatworm has been a pest to the coral aquaculture community for over a decade, it has only been found in aquaria and has never been described from the wild. Understanding its biology and ecology in its natural environment is crucial for identifying viable biological controls for more successful rearing of *Acropora* colonies in aquaria, and for our understanding of what biotic interactions are important to coral growth and fitness on reefs. Using morphological, histological and molecular techniques we determine that a polyclad found on *Acropora valida* from Lizard Island, Australia is *A. acroporae*. The presence of extracellular *Symbiodinium* in the gut and parenchyma and spirocysts in the gut indicates that it is a corallivore in the wild. The examination of a size-range of individuals shows maturation of the sexual apparatus and increases in the number of eyes with increased body length. Conservative estimates of abundance show that *A. acroporae* occurred on 7 of the 10 coral colonies collected, with an average of 2.6±0.65 (mean ±SE) animals per colony. This represents the first report of *A. acroporae* in the wild, and sets the stage for future studies of *A. acroporae* ecology and life history in its natural habitat.

## Introduction

The role of corallivory on coral reefs is becoming increasingly important to coral reef ecology given the number of other stressors coral reefs worldwide currently face [1]. Invertebrates are the majority of corallivores, outnumbering their fish counterparts nearly 3 to 1 [2], [3]. However, most invertebrate species have long been overlooked due to their small size and cryptic nature [4]. Corallivorous invertebrates may play an important role in coral health, inflicting minor or lethal damage on their coral hosts, which may subsequently have deleterious effects on coral growth and fitness [1]. They have also been implicated in transmitting or increasing vulnerability to coral disease [5], which indirectly contributes to coral loss or shifts in community composition. As scleractinian corals are the major reef builders, more attention is required to identify their predators and determine the roles they might play in maintaining or conserving coral reef ecosystems.

Two species of polyclad flatworms are known to prey on scleractinian corals [6], [7], yet very little is known about their impacts on coral reefs. As they are small and difficult to detect due to their excellent camouflage against the coral host, they may have been overlooked thus far in most studies of coral-associated animals. One such cryptic polyclad, the *Acropora*-eating flatworm (commonly known as the AEFW), was recently identified and classified as *Amakusaplana acroporae* Rawlinson et al., 2011 [7]. Known only from aquaria as a notorious pest of *Acropora* coral, this species has never been found in the wild. In fact, the taxonomic assignment was based on multiple specimens collected from two aquaria in the United States. Although most small animals that live and feed on corals have negligible, if any, ill effects on the coral host [8], infestations of *A. acroporae* on acroporids in captivity can result in rapid and complete colony death [9]. *A. acroporae* is a destructive predator of at least nine aquarium-reared Indo-Pacific acroporids (*Acropora valida, A. pulchra, A. millepora, A. tortuosa, A. nana, A. tenuis, A. formosa, A. echinata and A. yongei*), individuals lay multiple egg batches on an *Acropora* host and the hatchlings have a low dispersal capability [7]. These life history characteristics, combined with high prey specificity to *Acropora*, lend this species the potential to be a significant corallivore of *Acropora* corals.

Corallivory on *Acropora* corals is of particular interest to conservation management as *Acropora* is one of the most ecologically important coral genera to coral reefs worldwide. It is the largest extant coral genus, occurring in all tropical oceans as the dominant reef building coral [10]. Acroporids are a source of critical habitat and food for an immense diversity (∼150 species) of coral-associated animals [3], [11], [12]. They are extremely abundant and fast-growing branching corals yet are among the most susceptible corals to bleaching [13] and disease [14]. Furthermore, many corallivores actively select species of *Acropora* as their preferred prey [1], [2], such as the crown-of-thorns sea star, *Acanthaster planci* [15] and the gastropod *Drupella conus* [16]. Acroporids are also commercially important, being among the top three genera collected for the aquarium trade [17]. Thus acroporids are often the focus of conservation efforts, such as reef restoration [18], and an understanding of what biotic and abiotic interactions affect the growth, survival and distribution of acroporid corals is critical to their effective conservation.

Given *Amakusaplana acroporae*’s preference for Indo-Pacific *Acropora* species it is assumed that the worm is endemic to that region. Its cryptic coloration and relatively small size would make it difficult to detect *in situ,* hence its easy introduction into aquaria as *Acropora* epifauna. Locating *A. acroporae* in its natural environment would permit further study of its biology and ecological interactions, and this, in turn, could lead to the discovery of effective biological controls for this corallivore in captivity. This study aimed to determine whether an as-yet unidentified polyclad flatworm found on *Acropora valida* colonies from Lizard Island, Australia, was *Amakusaplana acroporae*.

## Materials and Methods

### Animal Collection and Fixation

**Figure 1:**
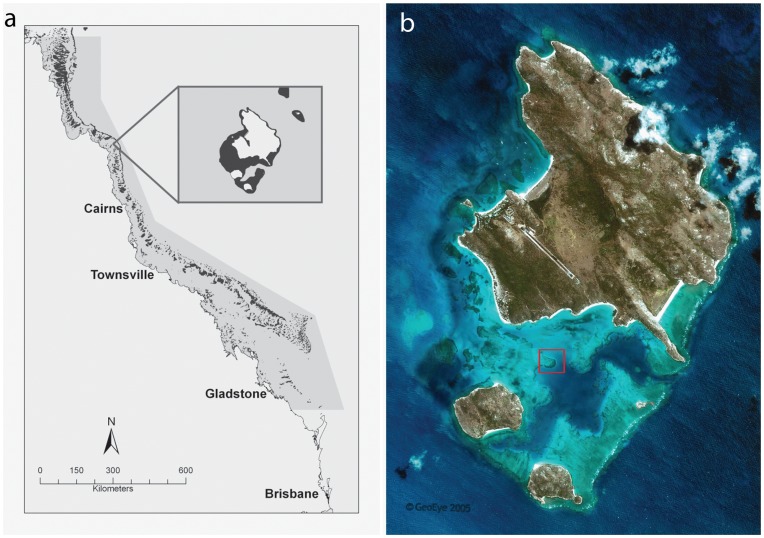
The collection site of *Amakusaplana acroporae.* (a) Map of the Northern Great Barrier Reef, Australia with inset of Lizard Island. (b) Photo of Lizard Island with collection site (red square) of *Amakusaplana acroporae* from its host coral *Acropora valida.* Photo credit “GeoEye satellite image”.

Animals were collected from Lizard Island, in the northern Great Barrier Reef, Australia (Fig. 1a) (under the Great Barrier Reef Marine Park Authority Permit: G09/32695.1). Sampling was conducted in November 2011, with average water temperatures ranging from 28.5–29.5°C. Ten colonies (ca. 20 cm diameter) of *Acropora valida* were collected at random from a shallow reef habitat (2–4 m depth) within the Lizard Island lagoon (Fig. 1b) (14°41′13.04 S, 145°27′20.06 E). All corals appeared to be in good health and did not show any signs of tissue damage. Coral colonies were first covered with a plastic bag to ensure animal retention, carefully chiseled off the substrate and transported in fresh seawater to the laboratory. Due to the cryptic nature of the polyclad associates, visual inspection did not yield any animals. Other macrofauna were visually identified and recorded. Corals were held over an empty container and the entire surface area, including all inter-branch space, was washed with high-pressured jets of seawater for approximately one minute. The water in the container was sieved through a 1×1 mm mesh, which was then inverted over a container of fresh seawater. This method proved to be successful at both dislodging the animals and maintaining them alive and in good condition. For histological and whole mount analysis, individuals were fixed on 4% frozen formaldehyde in seawater and left overnight at room temperature. Animals were then rinsed in seawater multiple times before being transferred to 70% ethanol for storage. For molecular analysis, adult specimens were preserved in 95% undenatured ethanol.

### Morphological Analysis

Histological and whole mount protocols are described in Rawlinson et al. [7]. For species identification paraffin-embedded histological sections (5 µM) were stained following a Masson’s trichrome protocol. The presence and distribution of *Symbiodinium* in the worm was confirmed by observing their autofluorescence with a Zeiss Axioscope fluorescent compound microscope on sections stained with DAPI (4′, 6-Diamidino-2-phenylindole, Sigma). Five individuals were sectioned in the transverse plane, three individuals were sectioned in the sagittal plane, and two individuals mounted as whole mounts. All material, including whole specimens, has been deposited in the Museum of Tropical Queensland.

### Molecular Analysis

Genomic DNA was extracted from one adult specimen (G20079) and the D1–D2 region of the 28 S rDNA gene was amplified using a novel forward (3′–5′) and reverse (3′–5′) primer pair designed for *Amakusaplana acroporae* based on conserved regions within aligned polyclad 28S rDNA sequences [7]. PCR was carried out using the following cycle temperatures/times: 4 min at 94°C; 45 cycles of 20 s at 94°C, 20 s at 52.5°C and 90 s at 72°C; 8 min at 72°C for a final extension. PCR was electrophoresed in a 1% agarose gel, and the product was excised and purified using the Qiagen MinElute Gel Extraction kit. The amplified fragment was cloned and sequenced in both directions using the pGem-T easy vector system (Promega). The 28 S rDNA D1-D2 region of G20079 (Genbank accession number JQ791553) was aligned using the ClustalW algorithm in MacVector with the polyclad sequences used in Rawlinson et al [7] (outgroup *Macrostomum lignano*). Phylogenetic trees were constructed using Bayesian Inference (BI) in MrBayes 3.2 [19]. The analysis was performed for 2,000,000 generations with a sampling frequency of 100. Node support was determined by posterior probabilities.

## Results

### Morphological Analysis

Analysis of the gross morphology was conducted on eighteen individuals, eight of which were sectioned for histological analysis of anatomy. We identified this animal to the family Prosthiostomidae (sub-order Cotylea) based on the following characters: absence of tentacles, a mouth at the anterior end of pharyngeal chamber, a tubular pharynx, a large muscular seminal vesicle adjacent to a pair of thick-walled accessory vesicles, a penis papilla and stylet enclosed in a penis pocket, a short vagina that is looped anteriorly and uterine canals arranged in an H-shaped figure [20]. Diagnosis to the genus *Amakusaplana* was established by the lack of a ventral sucker, a slight median depression in the anterior margin and irregularly scattered eyes in the anterior region of the body [21]. We determined that this animal is *Amakusaplana acroporae* (and not *Amakusaplana ohshimai*, the type and only other species of *Amakusaplana*) based on eye arrangement (distinct clusters of marginal and cerebral eyes in *A. acroporae*) and eye number (less than half the number of eyes in A. acroporae compared with *A. ohshimai*) and features of the reproductive systems (a bulbous female atrium and distinct egg chamber in *A. acroporae*) (see below and [7]).

**Figure 2:**
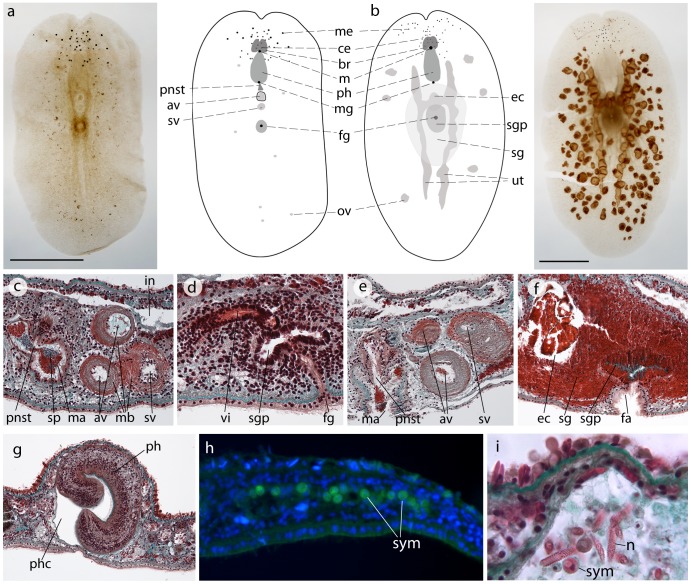
Anatomy and morphology of *Amakusaplana acroporae* from Lizard Island, Australia. Wholemounts and schematic representations of (a) a 3.2 mm and (b) a 5.0 mm long *A. acroporae* (scale  = 1 mm) showing gross morphology and development of the female reproductive structures. Individuals <4 mm in length possess (c) a mature male reproductive system, but (d) an immature female system. Individuals >4 mm in length possess mature (e) male and (f) female reproductive systems. (g) A cross section through the distal portion of the pharynx reveals its cleft morphology. *Symbiodinium* are present in the gut and parenchyma of *A. acroporae*, and may be observed (h) by autofluorescence and (i) light microscopy, spirocysts are also visible in the gut lumen. *av* accessory vesicle, *br* brain, *ce* cerebral eye, *ec* egg chamber, *fa* female atrium, *fg* female gonopore, *in* intestine, *m* mouth, *ma* male atrium, *mb* muscle bulb, *me* marginal eye, *mg* male gonopore, *ov* ovary, *ph* pharynx, *phc* pharyngeal cavity, *pnst* penis stylet, *sc* spirocysts, *sg* shell glands, *sgp* shell gland pouch, *sp* sperm, *sv* seminal vesicle, *sym Symbiodinium*, *ut* uteri, *vi vagina interna*.

Individuals of *Amakusaplana acroporae* collected from Lizard Island ranged in size from 3–6 mm in length and 1.5–3.5 mm in width when fixed. Examination of gross morphology and histological sections of animals with different body lengths revealed two trends in characters of taxonomic importance. Firstly, the number of eyes increases with body length. The two clusters of ventral marginal eyes increased from 5 eyes per cluster in a 3.2 mm long animal (Fig. 2a) to 10 eyes per cluster in a 5 mm long animal (Fig. 2b). The number of cerebral eyes clustered around the brain also increased from 27 to 35 in these two individuals (Fig. 2a & b). Secondly, the male reproductive system matures before the female reproductive system. The 4 individuals examined with a body length <4 mm had mature male but immature female reproductive systems. The male reproductive system consists of a penis armed with long scleratized stylet (Fig. 2c), which sits in the penis sheath and protrudes into the male atrium. The penis is connected via the ejaculatory duct to two accessory vesicles and a large seminal vesicle, each bound by a muscular sheath (Fig. 2c). Prostatic glands empty into the penis sheath and prostatic secretions and sperm are visible in the male atrium (Fig. 2c). While the female reproductive system in these individuals was immature, a female gonopore was present (Fig. 2d) but no eggs were visible in the uteri (Fig. 2a) and no shell glands were developed. Individuals ≥4 mm in length had mature male and female reproductive systems. Eggs were present in the ovaries and the paired uteri (Fig. 2b), well-developed shell glands surrounded the distended female atrium and distinct oval egg chamber (Fig. 2f), and sperm were present in the vas deferens and seminal vesicle. These developments in reproductive maturity with increased body length indicate that this animal is a sequential and then a simultaneous hermaphrodite.

*Amakusaplana acroporae* from Lizard Island differed from individuals collected from aquaria in two morphological traits. Firstly, in the number of marginal eyes clustered on each side of the anterior margin depression. Mature individuals from Lizard Island have 9.83±0.98 (mean ±SD; n = 6) marginal eyes per cluster instead of 2–3 in mature animals from aquaria. Secondly, when examined in cross section the tubular pharynx of *A. acroporae* is cleft [7]. This cleft appears only at the distal tip of the pharynx in the four animals examined in cross section from Lizard Island (Fig. 2g), whereas it extends further towards the gut in the specimen examined from captivity.

### Molecular Analysis

**Figure 3:**
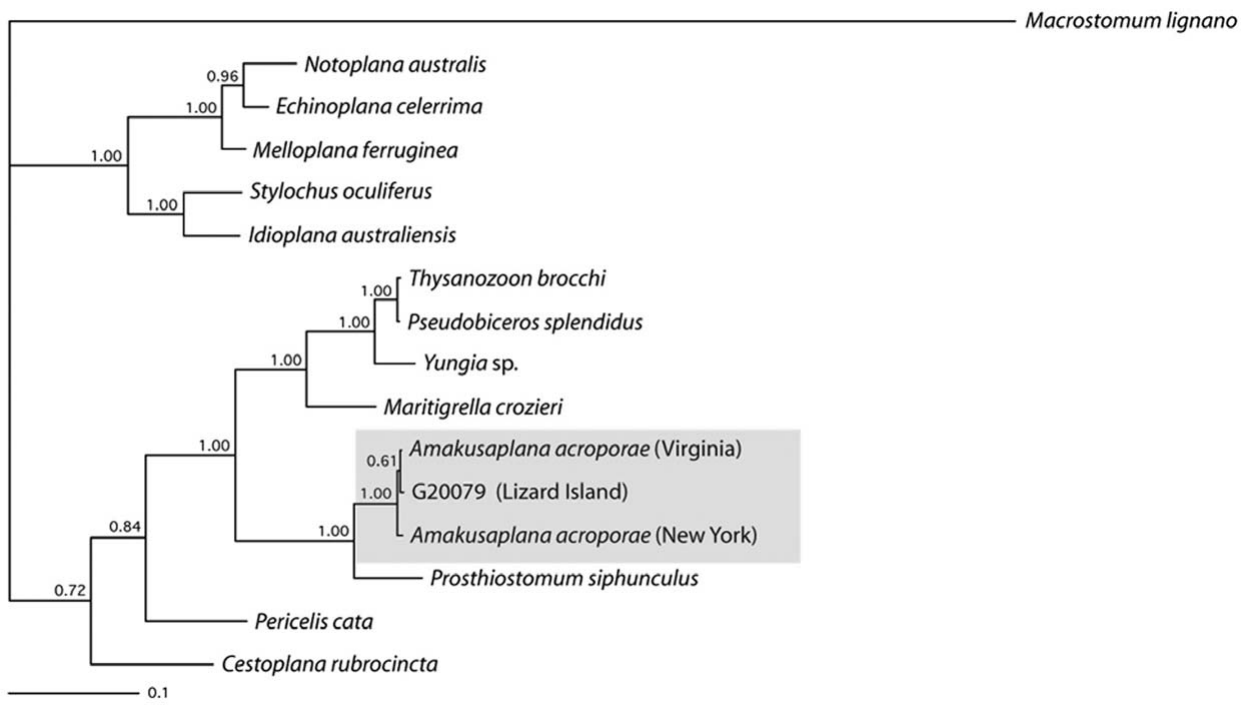
Consensus phylogenetic tree resulting from the Bayesian analysis of 28S rDNA sequence data. Clade support indicated by Bayesian posterior probabilities. The Lizard Island polyclad (G20079) falls out within a well–supported clade of *Amakusaplana acroporae* from captivity.

The Bayesian analysis of 28S rDNA sequence data (Fig. 3) resolves an individual from Lizard Island (G20079) to within the well supported clade (BI: 100%) of *Amakusaplana acroporae* collected from two different aquaria in the USA (Virginia and New York). This analysis is consistent with the morphology-based assignment of this individual to *A. acroporae.*

### Rates of Occurrence and Evidence of Corallivory

*Amakusaplana acroporae* occurred on 7 of the 10 coral colonies collected. Between 1 and 5 animals were found on each colony, with an average of 2.6±0.65 (mean ±SE) animals per colony. All eight individuals of *A. acroporae* that were examined histologically had *Symbiodinium* in the gut and parenchyma distributed throughout the body (Fig. 2h & i). The *Symbiodinium* were not observed intracellularly and their autofluorescence distinguished them from polyclad cells (Fig. 2h). Large (∼24 µm), unfired spirocysts were particularly abundant in the main intestinal trunk, less abundant in the intestinal branches and absent in the dorsal epidermis. Other amorphous material in the gut may have consisted of coral mucus and tissue.

### Other Macrofauna Present on the *Acropora Valida* Colonies

Each of the ten colonies contained other macrofauna, including a breeding pair of coral crabs (identified as *Tetralia nigrolineata)*, 2 gobies (*Gobiodon brochus*) and 2 palaemonid shrimp (*Coralliocaris graminea*).

## Discussion

This study identifies a polyclad flatworm found on *Acropora valida* colonies around Lizard Island as *Amakusaplana acroporae* and represents the first report of this animal in the wild. Evidence that *A. acroporae* is a corallivore in its natural habitat, as it is in aquaria, is supported by the presence of *Symbiodinium* and cnidarian spirocysts in the gut and parenchyma. In addition, the extracellular distribution of *Symbiodinium* implies that they were ingested and are not symbionts living within *A. acroporae*. Discovering *A. acroporae* in its natural environment and documenting a method of extracting the animals from their coral host alive will aid further research into the abundance, distribution and ecology of this corallivore.

Polyclad flatworms are morphologically quite homogeneous and over the past two centuries species descriptions and classifications have been based on a limited number of taxonomic characters [20], [22], [23]. These characters, used at all taxonomic levels, are described from the animal’s gross morphology and anatomy; for example the presence of a ventral sucker, the type and position of the pharynx, the presence of tentacles, details of the reproductive system and patterns of eyes. However, these last two sources of taxonomic characters, which are important for species level identification within the Prosthiostomidae, demonstrate plasticity during maturation as observed in this study and Kato [24] (in *Prosthiostomum (L.) purum)*. Therefore, without access to *Amakusaplana ohshimai* material for comparative morphological and molecular analysis we cannot rule out the possibility that *Amakusaplana acroporae* is synonymous with *A. ohshimai*, given that the characters that distinguish the two species (eye arrangement and number, morphometrics of the male and female reproductive systems and the presence of an egg chamber) vary with body length and maturation. This highlights the need to include in future species descriptions changes in morphological characters during development, and within and between populations, where possible. Nevertheless, from our morphological and molecular diagnoses we are confident that the polyclads collected from Lizard Island are the same species as that described from aquaria [7].

The presence of *Symbiodinium* and cnidarian spirocysts in the gut and parenchyma provides evidence that *Amakusaplana acroporae* is a corallivore in its natural habitat. No other prey items were observed in the gut of *A. acroporae* indicating that perhaps they are obligate corallivores (as has been demonstrated in the only other known scleractinian-eating polyclad *Prosthiostomum (Prosthiostomum) montiporae* [25]). As spirocyst morphology is fairly homogeneous within the Anthozoa [26] more direct evidence that *A. acropora* is feeding on *A. valida* would involve comparisons of molecular fingerprints of coral tissue in the gut contents with tissue from the coral host. Unlike some polyclad species that sequester nematocysts from their cnidarian prey in the lateral and posterior margins of their dorsal epidermis [27]–[29], there was no evidence of spirocysts being sequestered in *A. acroporae* in this study. While some corallivores have morphological adaptations that provide them with protections from coral nematocysts [30], how *A. acroporae* overcomes *Acropora* nematocysts is unknown.

As *Amakusaplana acroporae* is quite small, cryptic and possesses excellent camouflage against its acroporid coral host, this species is easy to overlook and thus far, their corallivory in the wild has probably been attributed to another species or even coral disease [30]. Moreover, until now this species has been unknown to marine ecologists, hampering any potential to learn about its role in coral health. Although Sweet et al. [31] reported that previous studies had found *A. acroporae* (or AEFW, as it would have been known at the time) in Indonesia and the Red Sea (citing [32]–[34]), Haapkylä et al. [33] actually refer to the acoel worm from the genus *Waminoa* which is a known coral-associate [35], and the other two studies do not mention flatworms. Although it is highly likely that the distribution of *A. acroporae* mirrors that of its *Acropora* species prey, and it could therefore be found in Indonesia and the Red Sea, visual surveying methods alone would probably not be sufficient to see *A. acroporae in situ* (Stella pers obs), although bite marks in the coral tissue and egg capsules on the bare coral skeleton might be visible on a heavily infested colony. As *A. acroporae* has been found in association with other Indo-Pacific *Acropora* species in aquaria (*A. pulchra, A. millepora, A. tortuosa, A. nana, A. tenuis, A. formosa, A. echinata and A. yongei* [7]), it is possible these species would be suitable natural hosts as well and might serve as a logical basis for learning more about these animals under natural conditions.

Gaining knowledge of the natural rates of occurrence and ecology of these polyclad worms will be vital to understanding its ecological role on coral reefs. No obvious tissue damage was evident on the *Acropora valida* colonies sampled in this study. That may, in part, be due to the small abundances (averaging less than three worms per colony) or the presence of natural predators within the coral colony. The estimates of abundance per colony in this study are somewhat underrepresented given that our sample size was small and the method was biased towards individuals greater than 1 mm^2^. *Amakusaplana acroporae* hatchling size is 250–300 µm [7] and these juvenile stages would have escaped collection. In aquaria some wrasse species have been observed to eat dislodged adult worms in the water column [7], [9]. Embryonic and hatchling life history stages may be vulnerable to a different set of predators, such as gastropods and decapods, which are highly diverse on acroporid corals [12]. Coral crabs, belonging to the genus *Tetralia,* have high occurrence rates on tightly branching acroporids [11] and are known to provide the coral host with cleaning services [36]. It is possible that these crabs may eat the adult worms and egg capsules, thus controlling the worms’ numbers. It is also possible that *A. acroporae* only becomes a serious pest in disturbed coral systems and aquarium environments, as is the case with *P. (P.) montiporae* [9], [25]. Further observations of *A. acroporae* in the field are needed to determine rates of coral tissue consumption (and subsequent colony mortality), identify its natural predators and quantify spatio-temporal patterns in its abundance.

Scleractinian corals are the most functionally important corals to reef processes, thus it is essential to understand what factors affect their growth and survival. Corallivores represent a biotic stressor that can detrimentally affect coral growth and fitness. In order to effectively manage conservation efforts of *Acropora* on coral reefs and to successfully rear colonies in aquaria, it is critically important to understand what biotic interactions are important to coral growth and fitness. This discovery of *Amakusaplana acroporae* in the wild and at Lizard Island will facilitate easy access to populations of this coral symbiont, enabling investigation of *A. acroporae* ecology, biology and life history in its natural habitat.
